# Maternal Nutrition Induces Pervasive Gene Expression Changes but No Detectable DNA Methylation Differences in the Liver of Adult Offspring

**DOI:** 10.1371/journal.pone.0090335

**Published:** 2014-03-03

**Authors:** Matthew V. Cannon, David A. Buchner, James Hester, Hadley Miller, Ephraim Sehayek, Joseph H. Nadeau, David Serre

**Affiliations:** 1 Genomic Medicine Institute, Lerner Research Institute, Cleveland Clinic, Cleveland, Ohio, United States of America; 2 Department of Genetics, Case Western Reserve University, Cleveland, Ohio, United States of America; 3 Pacific Northwest Research Institute, Seattle, Washington, United States of America; CNRS UMR7275, France

## Abstract

**Aims:**

Epidemiological and animal studies have shown that maternal diet can influence metabolism in adult offspring. However, the molecular mechanisms underlying these changes remain poorly understood. Here, we characterize the phenotypes induced by maternal obesity in a mouse model and examine gene expression and epigenetic changes induced by maternal diet in adult offspring.

**Methods:**

We analyzed genetically identical male mice born from dams fed a high- or low-fat diet throughout pregnancy and until day 21 postpartum. After weaning, half of the males of each group were fed a high-fat diet, the other half a low-fat diet. We first characterized the genome-wide gene expression patterns of six tissues of adult offspring - liver, pancreas, white adipose, brain, muscle and heart. We then measured DNA methylation patterns in liver at selected loci and throughout the genome.

**Results:**

Maternal diet had a significant effect on the body weight of the offspring when they were fed an obesogenic diet after weaning. Our analyses showed that maternal diet had a pervasive effect on gene expression, with a pronounced effect in liver where it affected many genes involved in inflammation, cholesterol synthesis and RXR activation. We did not detect any effect of the maternal diet on DNA methylation in the liver.

**Conclusions:**

Overall, our findings highlighted the persistent influence of maternal diet on adult tissue regulation and suggested that the transcriptional changes were unlikely to be caused by DNA methylation differences in adult liver.

## Introduction

Marked changes in human nutrition, behavior and lifestyle have resulted in escalating rates of obesity and type 2 diabetes mellitus [1]. While diet and lack of physical exercise constitute major risk factors for development of obesity and type 2 diabetes, intrauterine and early postnatal stresses also play an important role in the etiology of these diseases [2], [3]. In particular, maternal adiposity during pregnancy, and its associated comorbidities such as gestational diabetes, can permanently impact the health of offspring [4]. Several epidemiological studies have shown that maternal obesity is strongly associated with increased prevalence of obesity and type 2 diabetes in the progeny [5]–[10].

Several animal models have been developed to study the metabolic consequences of nutritional stress during development. For example, energy-rich diets during pregnancy in mice significantly influence body weight, hyperphagia, adiposity, insulin resistance and hypertension in offspring [11]–[13]. Rat models have also shown that intrauterine nutritional conditions can modify the response to an obesogenic environment in adulthood and increase the susceptibility to diet-induced obesity [14]–[16].

While these animal models have provided a thorough description of phenotypic and metabolic consequences of maternal nutrition in adult offspring, we still know little about the molecular mechanisms underlying these changes. Several studies have investigated molecular changes induced by maternal diets in a specific offspring adult tissue [17], [18] but no study has yet provided a global perspective on the molecular consequences of maternal nutrition on the adult offspring. In addition, while epigenetic mechanisms have been proposed to underlie developmental programming, few studies have tested this hypothesis and these were often limited by small sample sizes [19]–[26]. Here, we describe a mouse model that allows testing the molecular consequences of maternal nutrition on adult offspring and present genome-wide analyses of gene expression across tissues and detailed DNA methylation analysis in liver.

## Materials and Methods

### Animals

C57BL/6J mice were obtained from The Jackson Laboratory. Animals were housed in temperature-controlled rooms with 14–10 h light-dark cycling and *ad libidum* access to food and water. We only considered male offspring in all of the analyses described. Experimental procedures were approved by the Institutional Animal Care and Use Committees of Case Western Reserve University and the Cleveland Clinic.

### Experimental design

**Figure 1:**
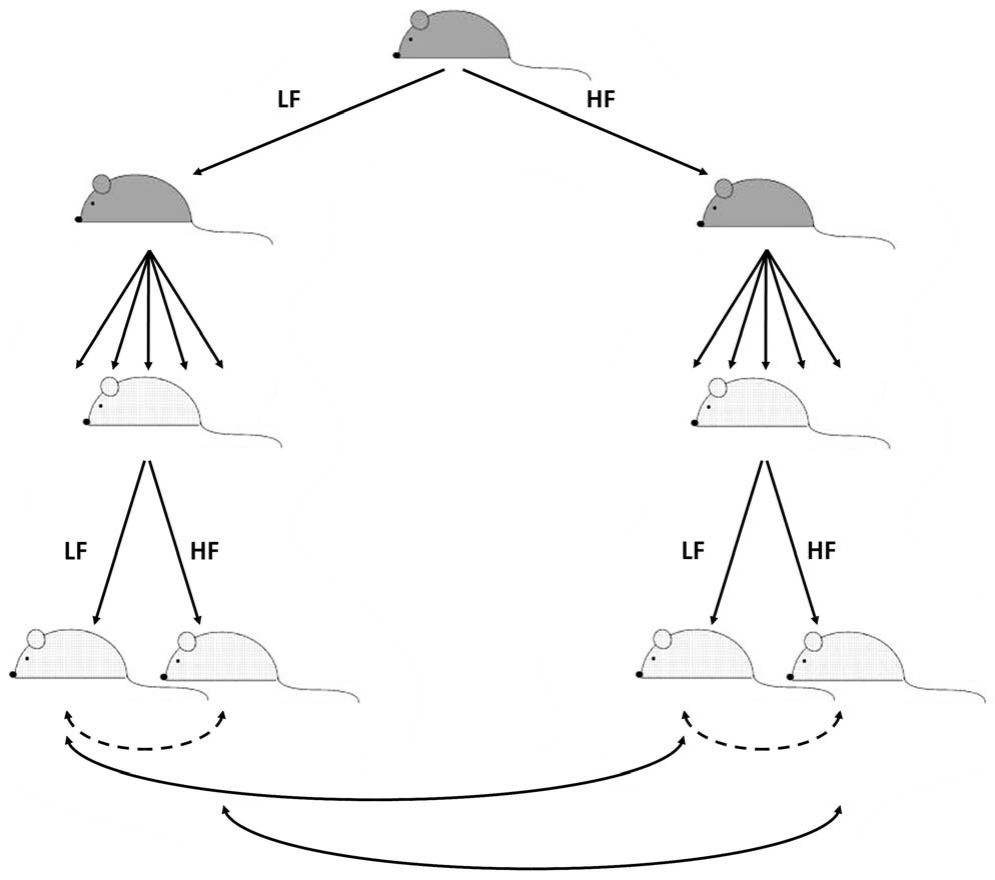
Overview of the experimental design. Dams are displayed in dark grey, offspring in light grey. LF and HF designate, respectively, low fat and high fat diet. Double-sided dashed arrows indicate pair-wise comparisons testing the effect of the diet post-weaning (on fixed maternal diet), solid arrows indicate the comparisons testing the effect of the maternal diet (on fixed post-weaning diet).

We assigned three-week-old virgin females to either a low-fat high-carbohydrate diet (LF, 10.5% kcal from fat) or a high-fat low-carbohydrate diet (HF, 58.0% kcal from fat). These diets (Research Diets Inc. D12328 and D12331) are nutritionally balanced, isocaloric, have identical quality and quantity of proteins (casein, 16.4% kcal) and have been previously used to study diet-induced obesity in C57BL/6J [27]–[30]. In both diets, the main source of fat is coconut oil. At eight weeks, one male was placed in one cage with two females and all animals were kept on the females' pre-mating diet. Males were removed after three days. After delivery, we equalized the number of pups per cage by randomly culling pups down to ten per cage if necessary. Animals from any litter with five or fewer pups were excluded from the study. Offspring were kept with their mother until weaning (day 21) when half of the male offspring from each cage were fed the HF diet and the other male offspring were fed the LF diet. Offspring were killed after a four-hour fast at either nine weeks of age (“Molecular Phenotyping Cohort”, n = 40) or six months of age (“Metabolic Phenotyping Cohort”, n = 47). The experimental design is summarized in **Figure 1**. For the rest of the manuscript, we refer to the maternal effect as the comparison of animals fed the same diet as adults, but whose mothers ate different diets (solid double arrows on Figure 1). The effect of the post-weaning diet refers to the comparison of animals fed different diets as adults but born from dams fed the same diet (dotted double arrow on Figure 1). Four male mice of the Metabolic Phenotyping Cohort that were hosted in a single cage, displayed enlarged hearts and abnormal blood glucose levels and were excluded from all analyses. For the Metabolic Phenotyping Cohort, we also followed seven male offspring born from LF fed dams whose diet was switched to HF upon delivery. These animals were all fed the HF diet post-weaning (**Figure S1**).

### Morphologic and metabolic phenotyping

After weaning, we killed all dams after a four-hour fast and measured fasting blood glucose and body weight.

For the Metabolic Phenotyping Cohort, we recorded the weight of each animal every week from weaning until 6 months of age. We measured blood glucose, for all animals (n = 47) after a four hour fast, at three time points (ten weeks, 21 weeks and six months of age) using a Bayer Contour glucose meter after nicking the animals' tail. For a subset of the animals (n = 5 for each of the four groups shown on **Figure 1**), we performed a Glucose Tolerance Test at 21 weeks. Fasting blood glucose was first measured at time 0. We then injected each mouse intraperitoneally with 2 mg of glucose per gram of body weight. After 30 minutes, blood glucose was re-measured and compared with glucose level at time 0. We also determined body composition and CO_2_ production using double-labeled water (n = 5 for each of the four groups) at five and a half months of age. CO_2_ production is an indirect measurement of energy expenditure and has been shown to correlate well with direct estimates. We injected each mouse with 2.25% of body weight in ml of double labeled water (3∶1 D_2_O:H_2_^18^O) and collected 25 ul of blood at 2, 26, 50, 74, 98 and 122 hours after injection. Samples were stored at -80°C until analysis. Samples were processed on a Agilent 5973N-MSD mass spectrometer equipped with an Agilent 6890 GC system and a DB-17MS capillary column (30 m×0.25 mm×0.25 um), and the data analyzed by ANOVA. At six months and after a four-hour fast, we collected blood by cardiac puncture from all animals. We then measured fasting blood glucose level and determined the concentration of fasting insulin and leptin in plasma (n = 49) with ELISA assays (Crystal Chem Inc., Downers Grove, IL, USA).

For the Molecular Phenotyping Cohort, we killed all offspring at 9 weeks of age and measured body weight, fasting blood glucose and fasting insulin and leptin in plasma as described above (**Table S1**).

### Gene expression profiling

For the Molecular Phenotyping Cohort, we collected in RNAlater (Ambion, Austin, TX, USA) samples of pancreas, skeletal muscle (thigh), white adipose tissues (gonadal pad), whole brain, liver and heart from 40 nine-week-old males (10 animals for each of the four combinations of maternal and post-weaning diet, **Figure 1**). We extracted total RNA using RNeasy purification kits (Qiagen Inc., Valencia, CA, USA) and hybridized each RNA sample to a MouseRef-8 v2.0 beadchip (Illumina Inc., San Diego, CA, USA). For each tissue, we quantile-normalized fluorescence signals from all 40 samples and only considered genes that were expressed in at least half of the samples.

We tested for differential gene expression between groups in pairs (**Figure 1**) using a Bayesian framework that models the dependence of the measurement variance on the level of gene expression [31]. We corrected statistical tests for multiple testing using false discovery rates (FDR) [32]. We performed pathway analyses with Ingenuity Pathway Analysis considering the fold-change expression of each differentially expressed gene (FDR<5%).

### DNA methylation profiling

We extracted DNA from the liver of all 9-week-old males (n = 40) using the DNeasy Blood and Tissue kit (Qiagen Inc., Valencia, CA, USA).

First, we characterized genome-wide patterns of DNA methylation in 8 liver samples by Methyl-CpG Binding Domain (MBD) protein isolation followed by genome sequencing as described previously [33]. Briefly, we sheared DNA samples to ∼200 bp and incubated them with protein G beads coated with recombinant MBD proteins. After washing, we eluted densely methylated DNA molecules to prepare Illumina sequencing libraries according to the manufacturer's instructions. We sequenced each library (n = 8) on one lane of a Genome Analyzer IIx to generate 27–35 million reads of 36 bp. We mapped all reads to the mouse reference genome sequence (mm9) using Bowtie [34]. We only considered reads mapped to a unique position in the genome and discarded putative PCR duplicates. We then determined which non-overlapping windows of the reference genome harbored significantly more reads than expected by chance (FDR<5%) using a Poisson distribution as previously described [33]. We conducted the analyses using either 100 bp and 1000 bp windows, as well as annotated CpG islands. We tested for qualitative differences in DNA methylation between pups born from HF and LF fed dams by looking for loci significantly methylated in all individuals from one group but lacking DNA methylation in all individuals from the other group. We also tested for quantitative differences between groups using the negative binomial framework implemented in EdgeR [35].

We also used 2 µg of DNA from each of the 40 liver tissues to characterize DNA methylation at individual CpGs using Reduced Representation Bisulfite Sequencing (RRBS) [36]. Briefly, we digested genomic DNA with MspI before preparing barcoded libraries according to the Illumina protocol. We then size selected DNA molecules of 150–600 bp and bisulfite converted the DNA libraries. 40 libraries (corresponding to 40 liver samples) were then sequenced on twelve lanes of a HiSeq 2000 to generate 5.8–33.9 million single-end reads of 50 bp per sample (mean = 14.3 million). We aligned all reads and determined the methylation status of each CpG for every read covering this position using Bismark [37]. We only analyzed individual CpGs that were covered by at least 10 reads in 90% of the samples and considered any CpG with less than 10 reads in any one sample as missing data. Lowering this analysis threshold would enable including more CpGs but would reduce our accuracy to determine the percent methylation and increase false positives (caused to high stochastic variations at lowly covered individual CpGs). We assessed statistical differences between groups using Student's t-test and Wilcoxon Rank test. We corrected all tests for multiple testing corrections using FDR.

Finally, we also bisulfite treated 2 µg of DNA from each of the 40 liver tissues using the MethylCode Bisulfite Conversion kit (Invitrogen, Carslbad, CA, USA) and PCR amplified 22 loci selected based on our gene expression analysis results. An overview of the experiment is presented in **Figure S2**. Briefly, we amplified each sample using 22 locus-specific primer pairs (**Table S2**) that include an identical 5′ oligonucleotide tail. After purification, we quantified the amplification products and pooled equal amount of all PCR products generated from a single sample. Each pool (corresponding to each individual sample) was then PCR amplified by a second set of primers targeting the 5′ oligonucleotide tail and containing the Illumina adapter sequence and a unique barcode sequence (**Table S2**). We then pooled all individually barcoded libraries together and sequenced the resulting library pool on one lane of Illumina HiSeq 2000. We determined, for each sample and each locus, the average proportion of cytosine methylation using Bismark and analyzed the data by the same method as described above for the RRBS data.

## Results

### Maternal diet influenced offspring growth and metabolism

To test the effect of maternal diet on growth and metabolism of the offspring, we assigned virgin C57BL/6J females to high fat (HF) or low fat (LF) diet five weeks before pregnancy and until day 21 postpartum (**Figure 1**). At mating and at postpartum day 21, dams fed the HF diet were significantly heavier than those fed the LF diet (Student's t-test, two-tailed, p = 4.8e-2 and p = 2.8e-2 respectively, **Table 1** and **Figure S3**).

**Table 1:** Influence of the maternal diet and diet after weaning on body size and metabolism regulation.

Dams				
Maternal diet	LF (±SE)	HF (±SE)	p Mat.	p Post
Body weight at mating (in g)	18.0 (±0.3)	18.9 (±0.4)	4.8e-2	
Body weight at postpartum day 21 (in g)	24.8 (±0.4)	26.5 (±0.7)	2.8e-2	
Fasting blood glucose at postpartum day 21 (in mg/dL)	190.1 (±39.0)	161.9 (±33.0)	0.14	
Litter size	7.0 (±0.5)	5.9 (±0.5)	0.15	

Offspring						
Diet after weaning	LF (±SE)	HF (±SE)	LF (±SE)	HF (±SE)		
Body weight at weaning (in g)	8.7 (±0.3)		10.1 (±0.2)		3.2e-4	
Body weight at 6 months (in g)	32.8 (±1.0)	48.8 (±2.0)	31.1 (±0.8)	41.1 (±1.4)	1.7e-2	3.8e-15
Body length at 6 months (in mm)	94.8 (±0.9)	99.6 (±1.1)	92.6 (±0.7)	96.2 (±1.0)	0.14	0.22
Fasting blood glucose at 10 weeks (in mg/dL)	117.6 (±6.2)	176.3 (±10.4)	131.5 (±5.1)	156.8 (±8.5)	0.25	0.93
Fasting blood glucose at 21 weeks (in mg/dL)	134.3 (±9.6)	193.0 (±7.7)	144.1 (±6.9)	179.9 (±10.2)	0.53	1.1e-6
Fasting blood glucose at 6 months (in mg/dL)	161.8 (±15.6)	208.8 (±11.7)	139.2 (±3.2)	175.9 (±6.5)	0.15	1.7e-4
Glucose tolerance test (change in glucose from 0-30 minutes)	193.2 (±21.7)	315.6 (±23.5)	191.8 (±38.0)	240.3 (±28.1)	0.35	5.7e-3
Fasting plasma leptin (in ng/mL)	5.0 (±1.4)	29.4 (±3.9)	6.0 (±1.2)	20.4 (±2.7)	0.27	0.27
Fasting plasma insulin (in ng/mL)	0.4 (±0.1)	2.0 (±0.3)	0.4 (±0.1)	1.1 (±0.1)	0.50	0.48
Fat mass (g)	9.6(±1.15)	26.33(±2.35)	11.49(±1.15)	17.82(±0.59)	0.74	0.86
Fat-free dry mass (g)	21.4(±1.5)	24.49(±1.09)	18.59(±0.64)	25.01(±1.86)	0.74	0.86
Energy expenditure (mol CO_2_/kg/day)	2.93(±0.26)	2.32(±0.3)	3.85(±0.3)	3.33(±0.39)	9.8e-3	3.0e-2

LF and HF stand for, respectively, low fat and high fat diet. p Mat and p Post indicate, respectively, the p-values associated with the influence of the maternal diet and diet after weaning. The influence of the maternal diet on dams' phenotypes and offspring's body weight at weaning was assessed using a Student's t-test. For all other offspring's phenotypes, the respective influence of the maternal and post-weaning diet was determined using a two-way ANOVA. After correcting for animal weight, many of the phenotypes did not reach statistical significance for the effect of post-weaning or maternal diet.

From most breeding cages, we obtained litters without significant differences between the two diet groups in litter size (Student's t-test, two-tailed, p = 0.15) or offspring sex ratio (Fisher's exact test, two-tailed, p = 1). We followed up a total of 21 male offspring from 6 HF cages and 26 male offspring from 5 LF cages. At weaning, male offspring from HF fed dams were significantly heavier than those born from LF fed dams (Student's t-test, two-tailed, p = 3.2e-4, **Table 1**). At this time, we separated the offspring and randomly assigned half of the males from each cage to either the HF or LF diet and maintained them on this diet until six months of age (**Figure 1**).

**Figure 2:**
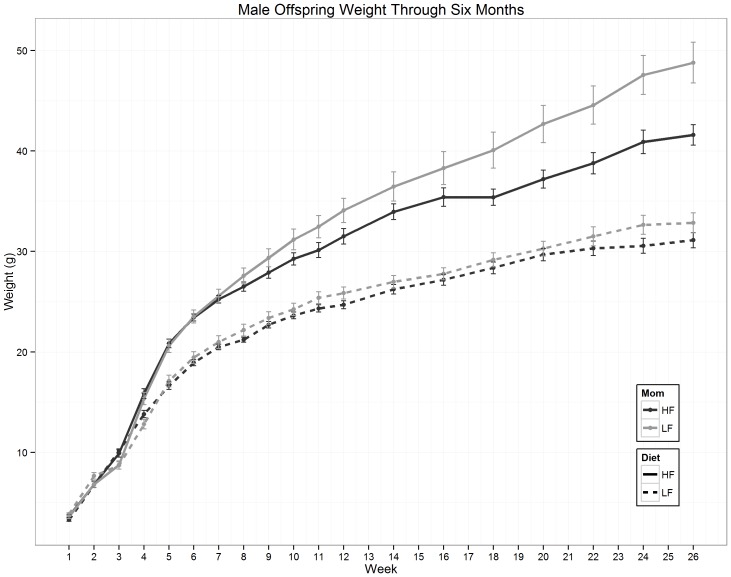
Body weight of male offspring. Average offspring weight (in g, ±SE) is presented from one week through six months of age. Maternal diet is denoted by line color (light gray: LF, black: HF) and adult diet is shown by line style (dashed: LF, solid: HF).

In six-month-old animals, we observed a significant effect of the post-weaning diet (i.e., comparing animals born from similarly fed dams, dotted arrows on **Figure 1**) on body weight and length (**Table 1** and **Figure 2**). While we did not detect any significant difference between animals fed the LF diet after weaning but born from mothers fed different diets, maternal diet significantly influenced weight gained by HF-fed offspring throughout their lives leading to a 17% body weight difference at six months (p = 7.3e-3, **Figure 2** and **Table 1**). Animals born from mothers fed a low fat diet but switched to a HF diet upon delivery had similar weights to animals whose mothers were fed a LF diet postnatally (**Figure S4)**. Glucose homeostasis did not seem affected by the maternal diet as illustrated by fasting blood glucose levels at ten weeks of age (n = 47, p = 0.25), 21 weeks (n = 47, p = 0.53) and six months (n = 47, p = 0.15) and results of glucose tolerance tests performed at 21 weeks of age (n = 18, p = 0.35) (**Table 1**). Plasma leptin and insulin were highly associated with body weight (n = 39, p = 9.46e-12 and <2e-16 respectively), but we did not detect any additional effect of the maternal diet (n = 39, p = 0.50 and 0.27, respectively) (**Table 1**). Double labeled water experiments showed that fat mass and fat-free dry mass were associated with body weight (n = 18, p = 4.3e-8 and 3.0e-4, respectively) (**Table 1**). Only daily energy expenditure (mol CO_2_/kg/day) was directly affected by the maternal diet (n = 17, p = 9.8e-3, **Table 1** and **Figure S5**): fat mass (n = 18, p = 0.74) or fat-free dry mass (n = 18, p = 0.74) were not significantly associated with maternal diet once adjusted for body weight differences (**Table 1**).

These results suggest that, in our mouse model, maternal diet predisposed the response to an obesogenic diet in male offspring and significantly influenced body weight and energy expenditure in adulthood, but had little effect on glucose homeostasis.

### Maternal diet had long-term influences on gene expression patterns of multiple offspring tissues

To identify molecular changes induced by the maternal diet in adult tissues and differentiate them from changes caused by the resulting body weight differences, we investigated tissues of 9-week-old mice that had similar weights irrespective of their dam's diet. Note that the phenotypes of the mice of this “Molecular Phenotyping Cohort” are very similar to those of the previously described “Metabolic Phenotyping Cohort” at the same age (**Table S1**).

We randomly selected ten nine-week-old males for each of the combinations of maternal and post-weaning diet (n = 40 total) and isolated RNA from each animal from pancreas, skeletal muscle, white adipose tissue, whole brain, liver and heart. We characterized the gene expression profile of each RNA sample by microarrays and used principal component analysis to identify possible outliers (**Figure S6**).

We determined which genes were significantly affected by the maternal or postnatal diet in a given tissue using pair-wise comparisons (**Figure 1**) and corrected for multiple testing by FDR. The results illustrate the complexity of interactions between the maternal and postnatal diets (**Table 2**). For example, when comparing animals fed different diets as adults, we noted that the diet after weaning significantly affected expression of ∼1,200 genes in liver regardless of the maternal diet. By contrast, maternal diet (comparing animals fed the same adult diet but born from mothers fed different diets) altered the expression of 158 liver genes when mice were fed LF after weaning and 634 genes when they were fed the HF diet (note that all comparisons have identical sample sizes). For most genes, the changes in expression level were modest – 1.2 to 1.6 fold – but highly significant due to the large number of biological replicates. The complete list of differentially expressed genes in all tissues is provided in **Table S3**.

**Table 2:** Results of the gene expression analysis.

Effect of the	Maternal diet		Post-weaning diet	
	when fed LF post-weaning	when fed HF post-weaning	when born from LF fed dams	when born from HF fed dams
Liver	158	634	1174	1251
WAT	393	323	1535	713
Muscle	537	85	653	289
Pancreas	422	306	742	374
Heart	198	352	341	336
Brain	0	129	7	271

The table shows the number of differentially expressed genes (FDR<5%) for each pairwise comparison and each tissue. The effect of the maternal diet is defined as the comparison of animals fed the same post-weaning diet, but derived from mothers fed different diets. The effect of the post-weaning diet is the comparison of animals fed different diets as adults and derived from mothers fed the same diet.

**Figure 3:**
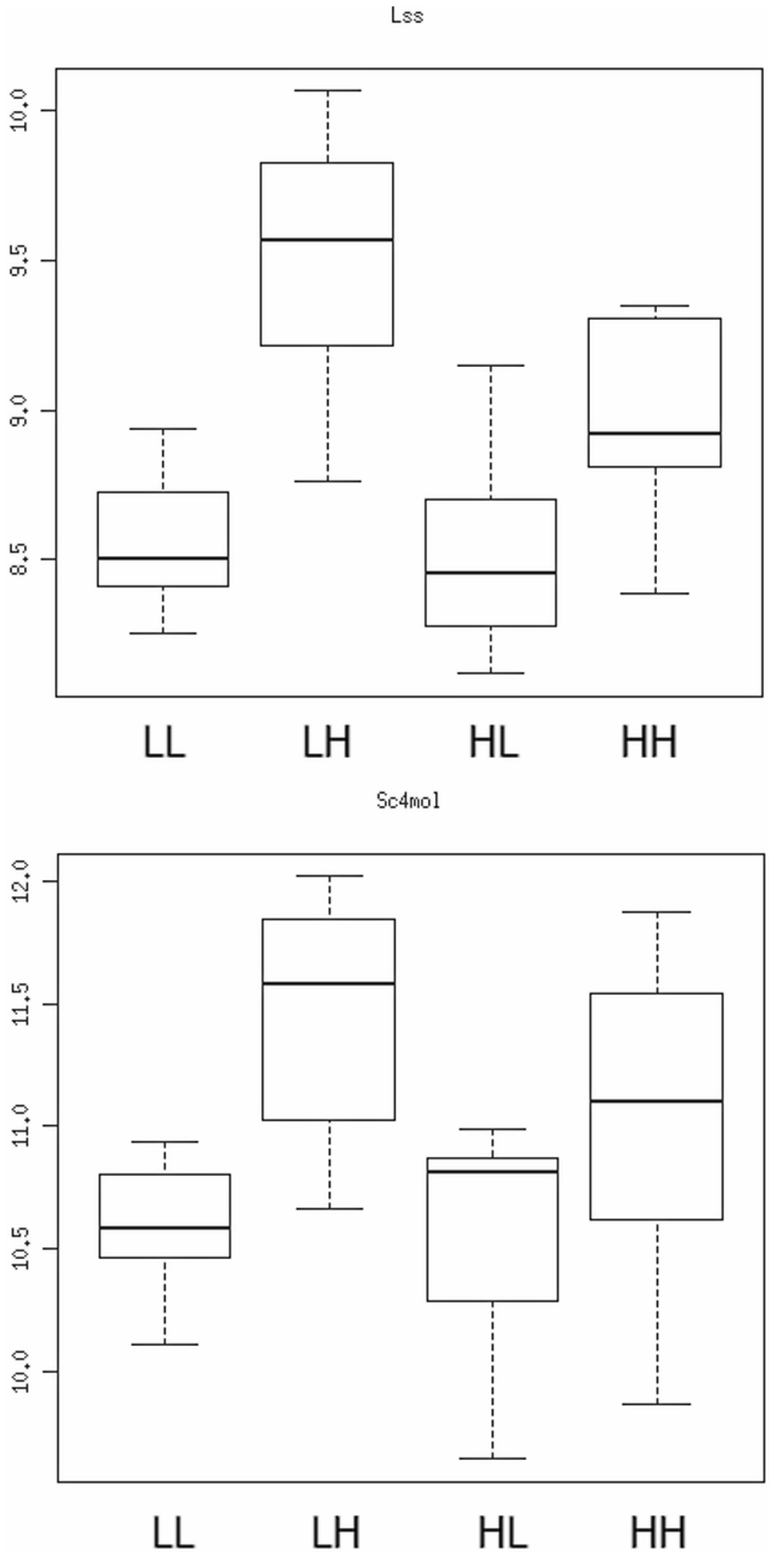
Expression level of two cholesterol synthesis genes in the liver of 9-week-old male offspring according to their maternal and post-weaning diets. The graph shows the normalized probe intensity measured for Lanosterol synthase (*Lss*, top panel) and Sterol-C4-methyl oxidase–like (*Sc4mol*, bottom panel) for all 40 samples. The dams' diet is indicated before the underscore, the post-weaning diet after it (the group order is identical to that of Figure 1). LF stands for low fat diet, HF for high fat diet. The thick line represents the median value of each group, the box delineates the 75^th^ percentiles, and the whiskers the 95^th^ percentiles.

**Figure 4:**
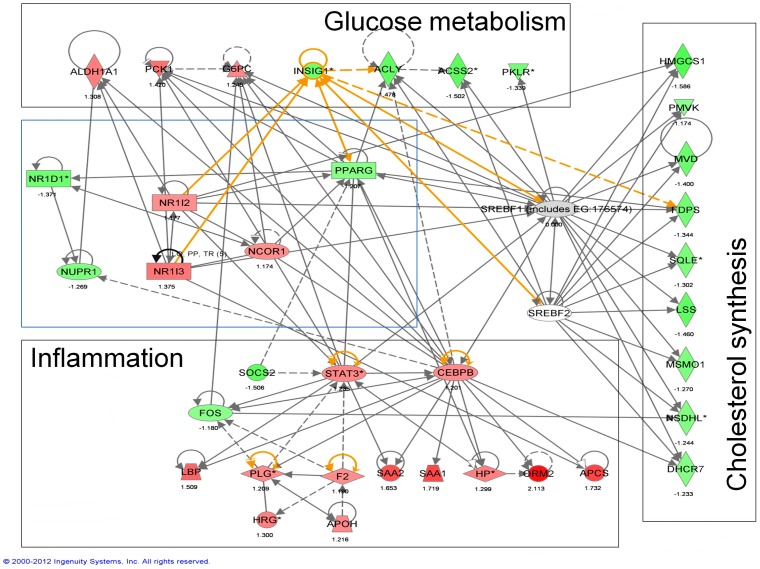
Summary of the gene expression patterns in liver. The figure displays some of the key molecular pathways affected by maternal diet (black boxes) with some representative genes. The blue box highlights some of the nuclear receptors that are differentially expressed according to the maternal diet and link Srebps and the most affected canonical pathways. Genes colored in green are over-expressed when the animals are born from LF fed dams (compared to animals born from HF fed dams), red genes that are under-expressed (with all animals being fed HF post-weaning).

Next, we investigated which molecular pathways were most influenced in adulthood by maternal diet. In liver, three canonical pathways were over-represented among genes whose expression levels were altered by maternal diet when offspring were fed HF after weaning: acute phase response signaling, biosynthesis of steroids and retinoid X receptor (RXR) activation (**Table S4**). Acute phase response signaling and RXR activation were also among the five pathways most affected by maternal diet in adipose tissue, pancreas and heart (but note that most genes differentially expressed according to the maternal diet are tissue-specific, **Figure S7.**). To understand how maternal diet affected these molecular pathways, we investigated in more detail the expression patterns of genes involved in cholesterol synthesis in liver. Fourteen genes involved in the synthesis of cholesterol from acetyl-CoA were significantly affected (FDR<5%) by maternal diet and thirteen showed a similar pattern: feeding the animals HF versus LF diet after weaning led to over-expression of these genes but the magnitude of this over-expression was greater when mice were born from LF fed dams (**Figure 3** and **Figure S8**). These patterns of expression changes suggested that maternal diet affected regulation of a key transcriptional regulator. Sterol regulatory element-binding proteins (Srebp) constituted obvious candidates since changes in regulation of these proteins lead to expression differences affecting similar genes as we observed [38]. *Srebf2* (the gene encoding for Srebp2) was not detected in our assay and diet did not significantly affect expression of *Srebf1* (encoding Srebp1). However, several genes known to interact with Srebp1 or Srebp2 [39], [40] had their expression modified by maternal diet. These included nuclear receptor genes, such as *Pparg*, CAR (*Nr1i3*), PAR (*Nr1i2*) or REV-ERBα (*Nr1d1*), acting with RXR and involved in homeostasis and metabolic regulation (**Figure 4**). Together with changes in *Cebpb* expression, changes in these nuclear receptors could explain most of the molecular pathways affected by maternal diet.

### No evidence of significant DNA methylation differences in adult liver samples

We tested whether the transcriptional changes in liver induced by maternal diet were associated with alterations of DNA methylation. First, we used high-throughput sequencing of DNA isolated after binding to MBD proteins to obtain a thorough characterization of the genome-wide patterns of DNA methylation in eight liver samples from 9-week-old animals, all fed the HF diet after weaning, and born from either LF fed dams (n = 4) or HF fed dams (n = 4). Our analyses identified 1–2% of the assembled mouse reference genome as densely methylated (FDR<5%), and these regions were similar across samples (**Figure S9–S10**). Indeed, of 662,451 100bp windows that were significantly methylated in at least one sample, none were consistently methylated in all samples from one group and lacked methylation in all samples from the other group. We also tested for quantitative differences in methylation between the two groups using all windows throughout the genome (i.e., not only those deemed to be significantly methylated) but did not identify any significant differences after correction for multiple testing. Analyses of different window-size (100 bp, 1000 bp or CpG island-based windows) yielded similar results (**Figure S11**). Note that while providing a true genome-wide perspective on DNA methylation, MBD-genome sequencing is probably semi-quantitative and may not be able to detect subtle difference in DNA methylation (see Discussion).

**Figure 5:**
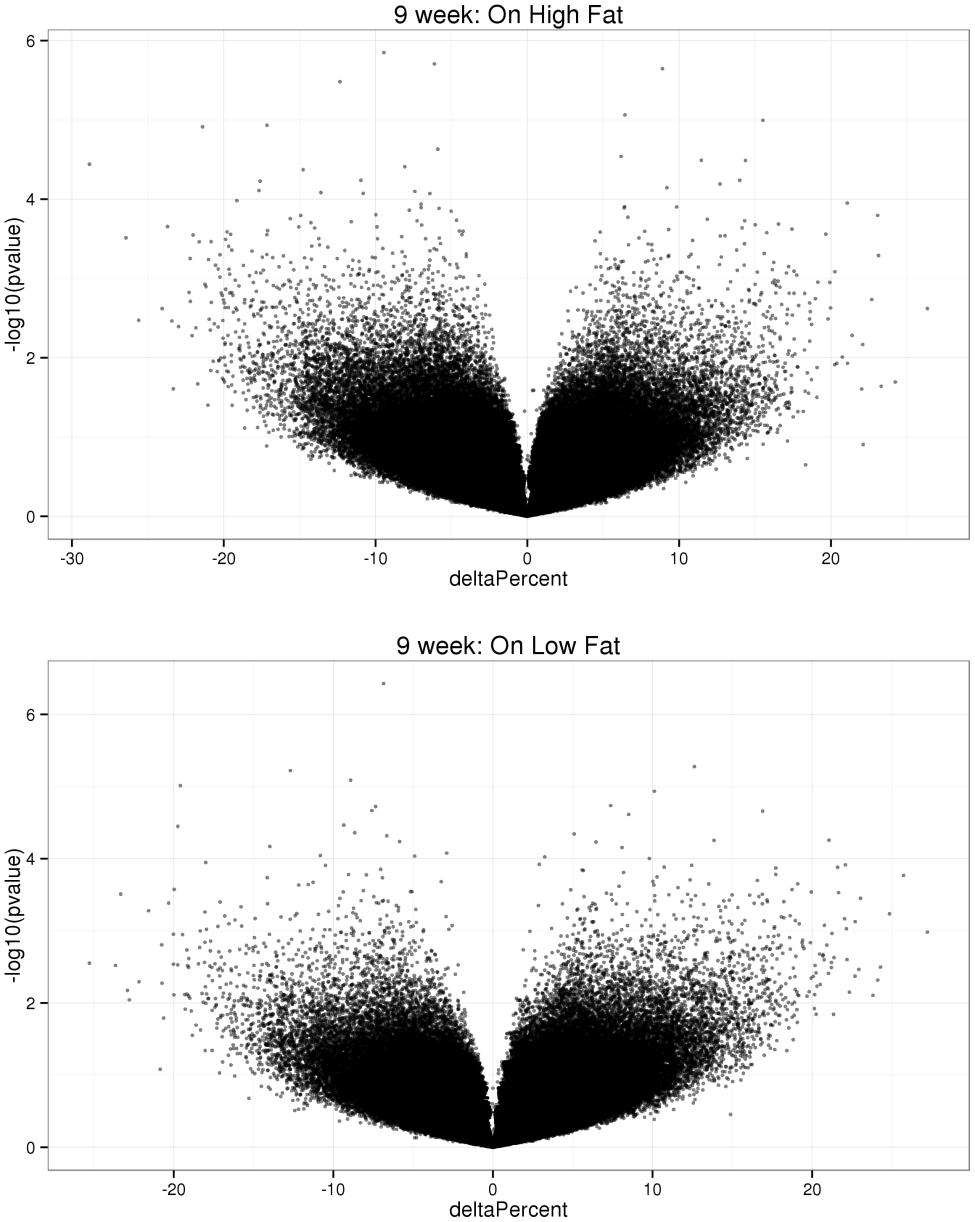
DNA methylation differences between 40 liver samples of 9-week-old male born from mothers on HF or LF diets. The figure shows DNA methylation differences at 400,298 CpGs between animals born from dams fed HF or LF diets (n = 10 per group). The x-axis shows the difference in average percent methylation between groups. The y-axis shows the –log10 of the p-value (uncorrected) associated with difference between groups. The top graph shows the results for animals fed HF diet after weaning, the bottom plot for animals fed LF diet after weaning adults. No difference is significant at a FDR of 5%.

We then used reduced representation bisulfite sequencing (RRBS) to obtain a quantitative characterization of the DNA methylation across a large fraction of the genome. After stringent quality controls, we were able to obtain robust assessment of DNA methylation at 400,298 CpGs for each of the 40 animals analyzed in the gene expression study. We did not detect any significant differences between animals born from mothers on different diets and maintained on the same adult diet (FDR <5%, **Figure 5**). Further simulations indicated that for CpGs with low intra-sample variability, we had more than 80% power to detect an 11% difference in methylation between groups (**Figure S12**). Relaxing our coverage threshold to include more CpGs yielded similar results.

Finally, to overcome the loss of power inherent to multiple testing correction in genomic studies, we analyzed DNA methylation at 22 loci selected based on our gene expression findings. We amplified bisulfite-converted DNA for each locus and each of the 40 male liver samples analyzed for gene expression and sequenced all PCR products simultaneously. Again, none of the loci investigated showed significant differences in DNA methylation at any individual CpG between groups after correction for multiple testing (**Figure S13**).

## Discussion

We described here a mouse model that enables studying the consequences of maternal nutrition. We showed that in our model, male mice born from LF fed dams gained significantly more weight than mice born from HF fed dams, but that this effect was only detectable if the animals were fed a HF diet after weaning. In addition, we showed that male mice born from LF fed dams had significantly lower energy expenditure than mice born from HF fed dams and fed a similar diet after weaning.

To begin understanding the molecular mechanisms underlying these phenotypic differences, we studied younger mice under the same experimental conditions. We hypothesized that by studying molecular changes at an earlier age, and particularly before the weight differences between groups become significant, we would enrich for pathways directly dysregulated by maternal diet rather than those affected by the resulting phenotypic differences. Our analyses revealed pervasive changes in gene expression associated with maternal diet across most tissues. The effect of the maternal diet on gene expression was typically modest (∼1.2 fold in expression level), but statistically significant due to the large number of biological replicates and consistency (in direction and intensity) across genes within a molecular pathway (**Figure S8**).

The effect of the maternal diet on gene expression was most pronounced in liver, where more than 5% of the genes were affected. The gene expression changes induced by maternal nutrition depended heavily on the offspring's diet after weaning, mirroring the phenotypic patterns observed in 6-month-old animals. The number of genes affected by maternal diet was four-fold greater when the animals were fed HF diet after weaning compared to animals fed LF diet. In addition, many genes influenced by the maternal diet were also affected by the post-weaning diet. For example, cholesterol synthesis genes displayed higher expression in animals fed HF than LF diet, and the magnitude of this difference depended on the diet of their dams: animals exposed to high fat during development presented a lesser response to HF diet after weaning (**Figure 3**). These observations suggest that, in our model, HF diet during pregnancy “protects” male offspring from diet-induced obesity. This effect contrasts with other studies of maternal obesity that often show the opposite effect [11]–[13]. One major difference between such studies and ours is the source of fat (lard vs. coconut oil) resulting in dramatic nutrient differences. For example, coconut oil contains 86.5% saturated fatty acids (primarily 12 carbon lauric acid) while lard contains only 39.2% (and primarily 16 carbon palmitic acid) (****, data from http://ndb.nal.usda.gov/). Coconut oil also contains less than 6% (wt/wt) monounsaturated and 1.8% polyunsaturated fatty acids, compared to 45.1% and 11.2% for lard. In addition, lard contains 95 mg of cholesterol which is absent in coconut oil. Composition in choline and various vitamins also differs between coconut oil and lard. It is possible that mice respond differently to intrauterine exposure to different fatty acids or other nutrients present in the two diets but further research will be required to better understand the specific role of these nutrients.

In our setting, many of the gene expression changes induced by maternal diet in liver could result from a primary/upstream change in Srebp regulation [38]. We did not observe transcriptional differences in *Srebf1* or *Srebf2*, but cannot rule out that these proteins were directly responsible for the patterns observed since they are primarily regulated post-transcriptionally [41], [42]. Interestingly, we observed significant changes in the expression level of several key metabolic transcription factors such as Pparg, CAR, PAR or REV-ERBα. Several of these nuclear receptors influence the Srebp pathway through binding of retinoic acid [40], [43], [44]. This observation is further supported by our pathway analysis which highlighted RXR-activation as one of the canonical pathways most affected by maternal diet in liver (and several other adult tissues). Based on the results of our gene expression analyses, we hypothesize that the consequences of maternal nutrition are mediated by lasting dysregulation of RXR-regulated pathways.

We attempted to identify the primary molecular changes induced by maternal diet and tested whether epigenetic mechanisms could be responsible for the pervasive changes in gene expression. We focused on DNA methylation which is established during development and tends to be responsible for long-term gene expression silencing, while histone modifications have a more dynamic role [45], [46]. Our genome-wide characterization of the DNA methylation marks in liver using MBD isolation genome sequencing did not reveal any significant epigenetic differences induced by the maternal diet. This result indicates that maternal diet does not lead to dramatic differences in DNA methylation patterns in adult liver (such complete loss or gain of methylation at a given locus). While this analysis enables a truly genomic characterization of DNA methylation, it suffers from the small number of samples and is only semi-quantitative. In addition, MBD proteins only bind to densely methylated DNA fragments and do not efficiently capture isolated methylated CpGs. This analysis would therefore miss changes in DNA methylation at isolated CpGs. We thus complemented this study with a quantitative assessment of DNA methylation at >400,000 CpGs throughout the genome using all 40 liver samples. Again, we failed to detect any significant differences in DNA methylation associated with maternal diet. Finally, to overcome the multiple-correction burden inherent to genomic studies, we characterized DNA methylation for all 40 liver samples at 22 selected loci using bisulfite sequencing. This approach also failed to identify any significant differences in DNA methylation in the regions assayed by these techniques. Several reasons may explain these findings. First, it remains possible that we failed to identify localized changes in DNA methylation that occurred at isolated CpGs or in other regions of the genome that were not investigated by any of our three approaches. Second, we characterized DNA methylation patterns in liver where we observed most of the transcriptional changes induced by maternal diet (quantitatively). It is possible that maternal diet induced epigenetic changes in another organ and that the gene expression changes observed in the liver only occurred as a consequence of primary changes elsewhere. Third, it is possible that epigenetic alterations occurred during development but were erased with time while their consequences on gene expression remained through nine weeks (e.g., controlled by feedback mechanisms). Fourth, we analyzed genomic DNA extracted from whole liver and the DNA methylation patterns therefore likely represent the epigenetic marks of hepatocytes (which constitute the overwhelming majority of the liver). It is possible that DNA methylation changes occurred in another cell population (e.g. Kupffer cell) and were missed by our analyses (note however that most gene expression changes associated with maternal diet were related to metabolism and unlikely to be caused by dysregulation on these minor cell populations). Finally, it is possible that the translation of maternal nutritional stress into molecular mechanisms in our model is independent of DNA methylation.

A limitation of our study is the difficulty in separating the pre-conception, *in utero*, and early postnatal effects of the maternal diet. These are confounded in our experimental design: dams were fed the same diet for 5 weeks before mating, throughout pregnancy and until day 21 postpartum. To preliminarily assess these effects, we analyzed a small group of animals whose dams were switched from a LF diet to HF diet upon delivery. The adult animals were phenotypically similar to animals born from mothers fed a LF diet throughout pregnancy and lactation, which suggests that most of the phenotype changes we observed were indeed induced by intrauterine effects.

Overall, our experimental design and the findings presented here provide a framework to better understand the molecular mechanisms induced by maternal nutrition. Future studies of this animal model could identify promising pharmacological avenues to overcome developmental programming and could contribute to reduce the risk for offspring to develop obesity.

### Data availability

MBD data can be found under SRA# GSE52266. Microarray data are available on the GEO database (GSE40897, GSE40898, GSE40899, GSE40900, GSE40901, GSE40902, GSE40903). RRBS and locus specific bisulfite sequencing are available on the GEO database (GSE52268).

## Supporting Information

Figure S1 **Experimental design with dams whose diet was switched at birth.** Dams are displayed in dark grey, offspring in light grey. LF and HF designate, respectively, low fat and high fat diet. Females on the left were fed a LF diet until birth when the diet was switched to HF until postpartum day 21 (weaning).
(TIF)

Figure S2 **Description of locus specific bisulfite PCR library protocol.** Samples are first amplified by multiple locus specific PCR primers in separate reactions (“Locus specific PCR”) and subsequently pooled. Adapter and barcoding sequence are added to each pool in a second round of PCR (“barcoding PCR”). After barcoding, the libraries are pooled into a single tube and sequenced.
(TIF)

Figure S3 **Maternal weight.** Average weight (in g, ±SE) of dams is presented from three weeks of age through weaning. Mating at eight weeks and weaning are noted.
(TIF)

Figure S4 **Male offspring weights for all five groups of animals.** Animal weights in grams (±SE) are presented from one week through six months of age. Animals are separated by maternal and adult diets. The maternal diet labeled “LowSwitch” represents the cohort of animals for which the dams' diet was changed from LF to HF upon delivery and offspring were fed on a HF diet after weaning.
(TIF)

Figure S5 **Energy expenditure in adult offspring is influenced by maternal diet.** Energy expenditure (mol CO_2_ produced per day per kg of animal body weight) is increased in male offspring of mothers fed a high fat diet during pregnancy.
(TIF)

Figure S6 **Principal component analysis of the gene expression patterns across tissues.** Each dot represents one tissue from one male mouse. Red dots represent pancreas samples; light blue, liver samples; yellow, adipose tissues; green, brains; dark blue, muscle and pink, heart.
(TIF)

Figure S7 **Heatmap of gene expression data for six tissues for the comparison of animals fed a high fat diet post-weaning, but from mothers fed a different diet.**
(TIF)

Figure S8 **Liver gene expression level for 14 genes involved in cholesterol synthesis.** See legend of Figure 3 for details.
(TIF)

Figure S9 **Comparison of the genome-wide DNA methylation patterns obtained by MBD-Genome sequencing of two liver samples.** Each dot of the graph represents one 100 bp window of the genome and is displayed according to the number of reads obtained after MBD isolation in the liver sample of a male born from a HF fed dams (x-axis) and a male born from a LF fed dam (y-axis) (both mice being fed HF post-weaning).
(TIF)

Figure S10 **Comparison of the DNA methylation patterns obtained by MBD-GS across ∼200 kb in four liver samples.** The figure shows the DNA methylation patterns across 200 kb of chromosome 6. The top track shows in blue the genomic coordinates of genes based on the UCSC annotation. The next tracks show the methylation patterns observed from MBD isolation and genome sequencing for two liver samples from males born from HF fed dams (top two tracks) and two liver samples from males born from LF fed dams (bottom two tracks). Each brown vertical line display the number of reads obtained per 100 bp window for each sample.
(TIF)

Figure S11 **Volcano plots of MBD-GS results.** The volcano plots show the fold change in MBD read counts (x-axis) and associated p-value (–log10(uncorrected p-value, y-axis) for each tested genomic locus (window sizes are indicated above the volcano plots). After FDR correction, no windows were significantly differentially methylated.
(PNG)

Figure S12 **RRBS Power Analysis.** The figure shows the statistical power (y-axis) to detect a given difference in DNA methylation (x-axis) at conserved CpGs. To estimate power, we randomly sampled means and standard deviations from the RRBS data generated for the HH group (n = 10) and simulated RRBS data for two groups (each n = 10) with a fixed average difference in DNA methylation (x-axis). 10,000 simulations (i.e. 10,000 random selections of mean and SD) were performed for each fixed difference in methylation and the percent of significant tests (p<0.05) calculated. Only CpGs with low DNA methylation variability within group (lowest 10% of standard deviation) were used in this analysis (the results are qualitatively similar using the entire dataset). The red line represents 80% power.
(TIFF)

Figure S13 **DNA methylation patterns at 22 loci using bisulfite sequencing.** The figure shows the average cytosine methylation of each CpG of 22 selected loci after bisulfite sequencing of 20 liver sample from 9-week-old offspring fed HF after weaning and born from LF fed dams (in blue, n = 10) or HF fed dams (in red, n = 10).Each plot corresponds to a different locus. The y-axis shows the average DNA methylation (for 0 to 100%). The x-axis shows each CpG sequenced in a given locus. No difference remains significant after correction for multiple testing.
(TIF)

Table S1 **Comparison of the offspring phenotypes for the “Molecular phenotyping” and “Metabolic phenotyping” cohorts at nine weeks.** LF and HF stand for, respectively, low fat and high fat diet. p Mat and p Post indicate, respectively, the p-values associated with the influence of the maternal diet and diet after weaning. The influence of the maternal diet on dams' phenotypes and offspring's body weight at weaning was assessed using a Student's t-test. For all other offspring's phenotypes, the respective influence of the maternal and post-weaning diet was determined using a two-way ANOVA.
(XLSX)

Table S2 **Primer sequences used for locus specific bisulfite sequencing.** For primers used in the first round of PCRs, the primers were the combined sequences of the 5′ tail and priming sequence. Barcodes present in second primers are noted beside the primer sequences.
(XLS)

Table S3 **Lists of the differentially expressed genes in each tissue and for each comparisons.**
(XLS)

Table S4 **Molecular pathways overrepresented in the differentially expressed genes.** The table shows the five most differentially regulated pathways for each tissue and comparison based on Ingenuity Pathways Analysis. The comparisons made reveal the influence of either the maternal diet or post-weaning diet. These comparisons are broken down further by either post-weaning or maternal diets. The maternal diet and post-weaning diet are presented in a two letter code, with the first letter indicating the maternal diet, and second the post-weaning diet.
(XLSX)

Table S5 **Nutritional content comparison of coconut oil and lard. Data were obtained from** http://ndb.nal.usda.gov/.
(XLSX)
