# Supplementary figures and images for: Maternal Nutrition Induces Pervasive Gene Expression Changes but No Detectable DNA Methylation Differences in the Liver of Adult Offspring
Source: PLoS One. 2014 Mar 3;9(3):e90335. doi: 10.1371/journal.pone.0090335 (PMC3940881; doi:10.1371/journal.pone.0090335)

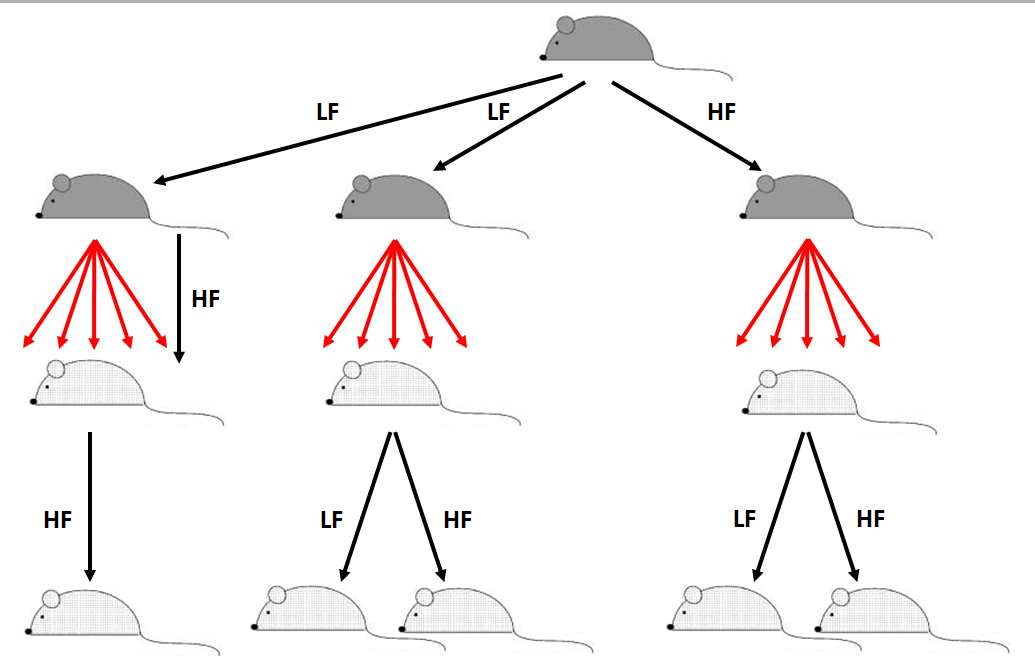

Supplement: Figure S1 — Experimental design with dams whose diet was switched at birth. Dams are displayed in dark grey, offspring in light grey. LF and HF designate, respectively, low fat and high fat diet. Females on the left were fed a LF diet until birth when the diet was switched to HF until postpartum day 21 (weaning). (TIF) [file pone.0090335.s001.tif]

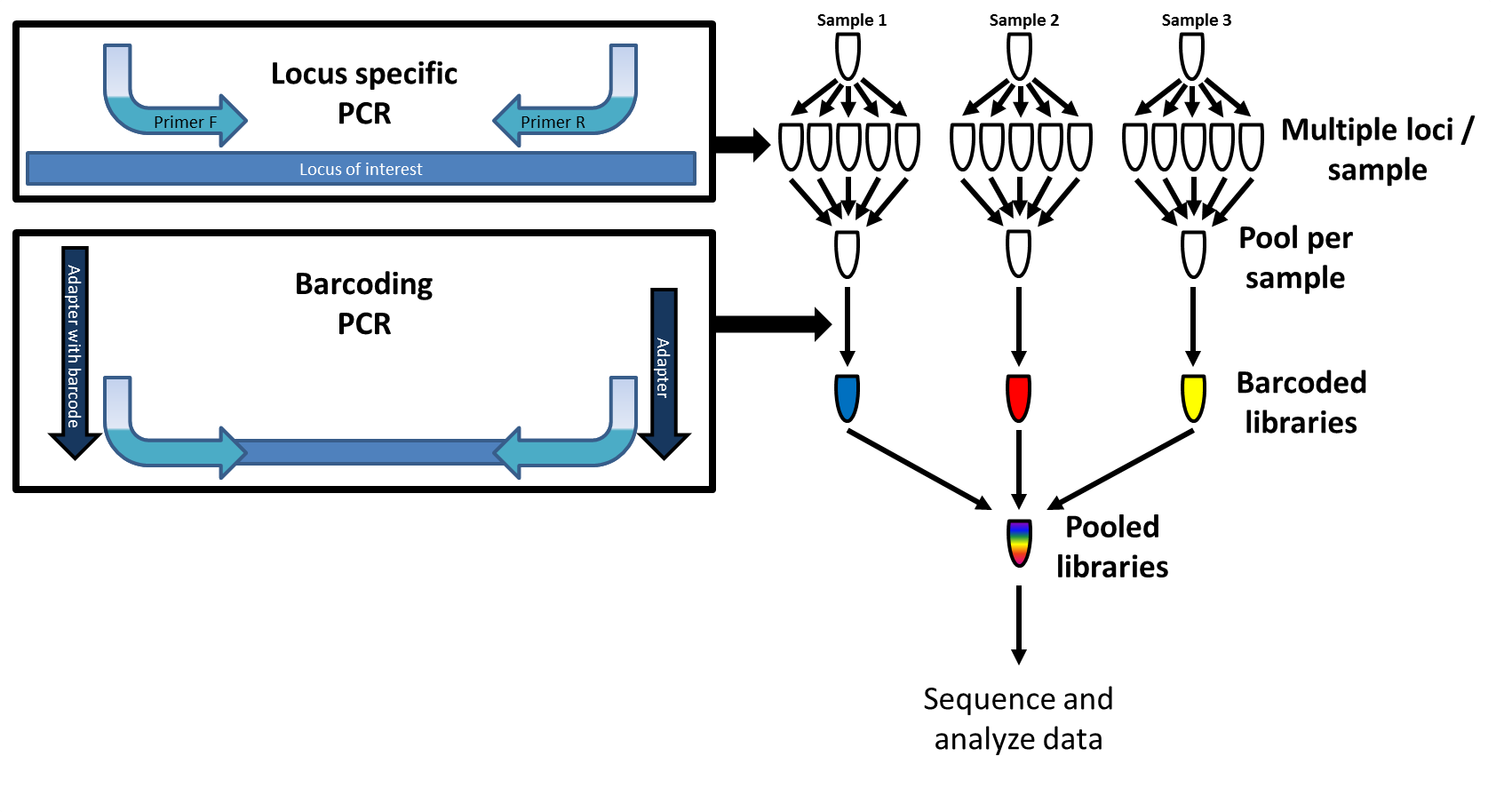

Supplement: Figure S2 — Description of locus specific bisulfite PCR library protocol. Samples are first amplified by multiple locus specific PCR primers in separate reactions (“Locus specific PCR”) and subsequently pooled. Adapter and barcoding sequence are added to each pool in a second round of PCR (“barcoding PCR”). After barcoding, the libraries are pooled into a single tube and sequenced. (TIF) [file pone.0090335.s002.tif]

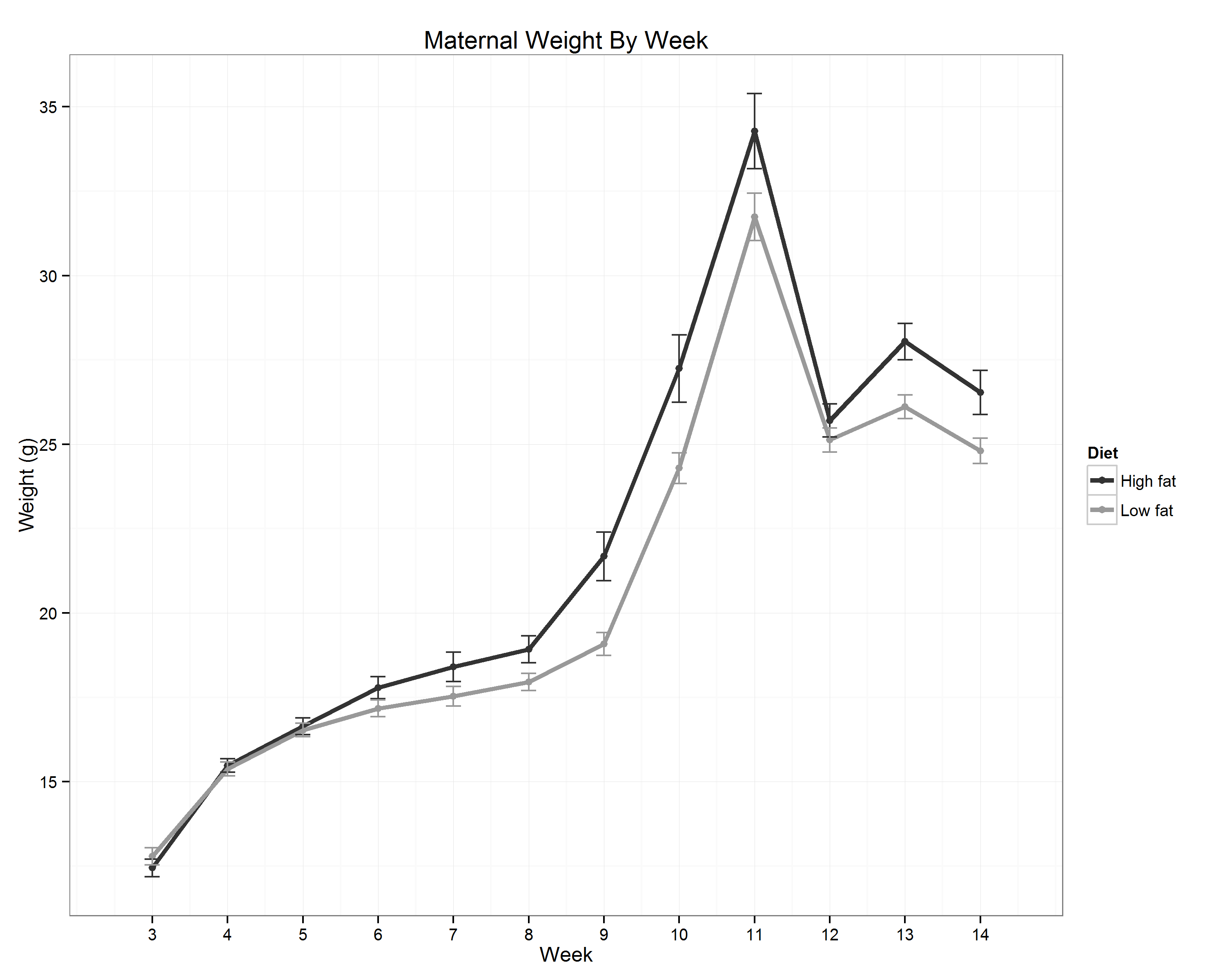

Supplement: Figure S3 — Maternal weight. Average weight (in g, ±SE) of dams is presented from three weeks of age through weaning. Mating at eight weeks and weaning are noted. (TIF) [file pone.0090335.s003.tif]

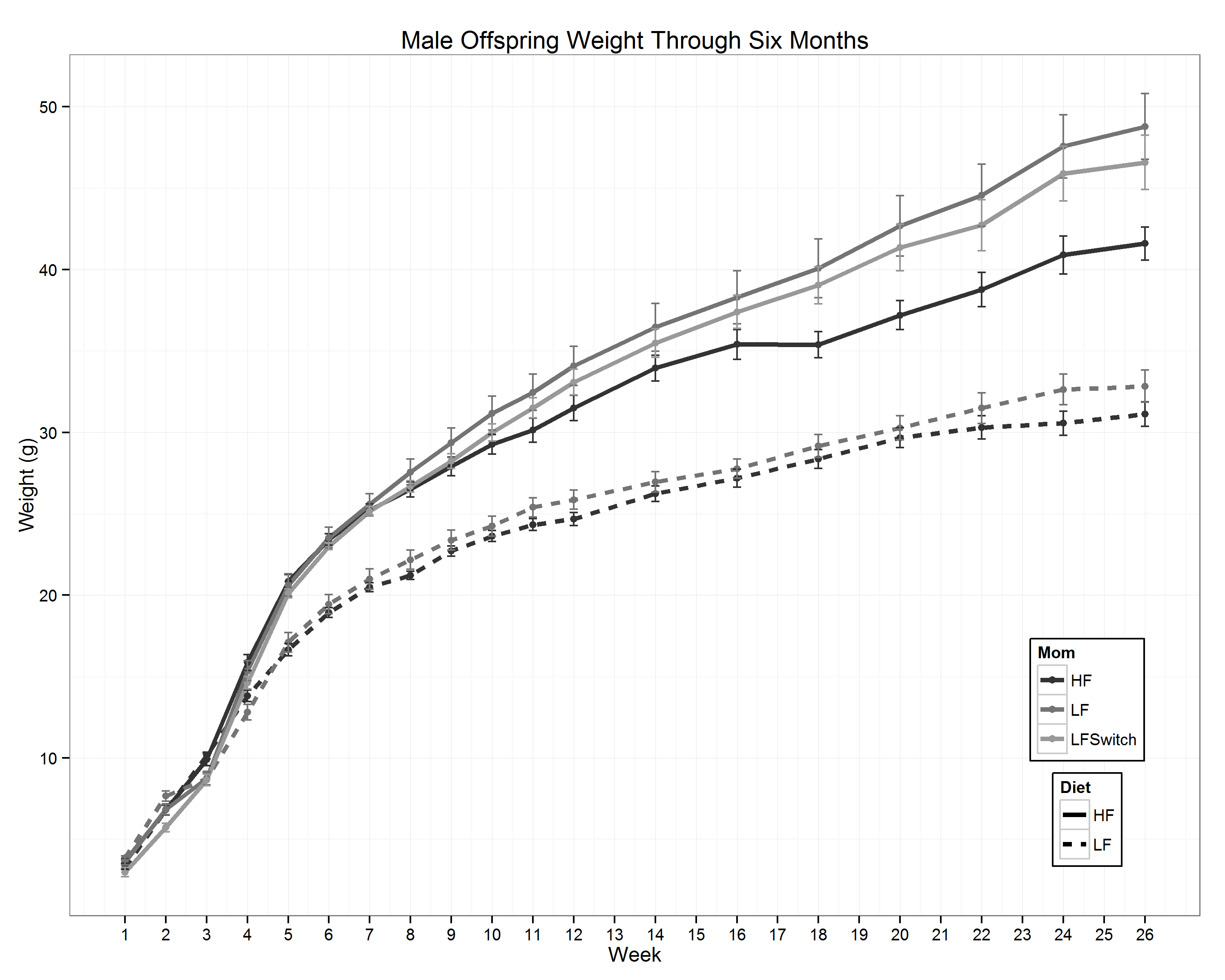

Supplement: Figure S4 — Male offspring weights for all five groups of animals. Animal weights in grams (±SE) are presented from one week through six months of age. Animals are separated by maternal and adult diets. The maternal diet labeled “LowSwitch” represents the cohort of animals for which the dams' diet was changed from LF to HF upon delivery and offspring were fed on a HF diet after weaning. (TIF) [file pone.0090335.s004.tif]

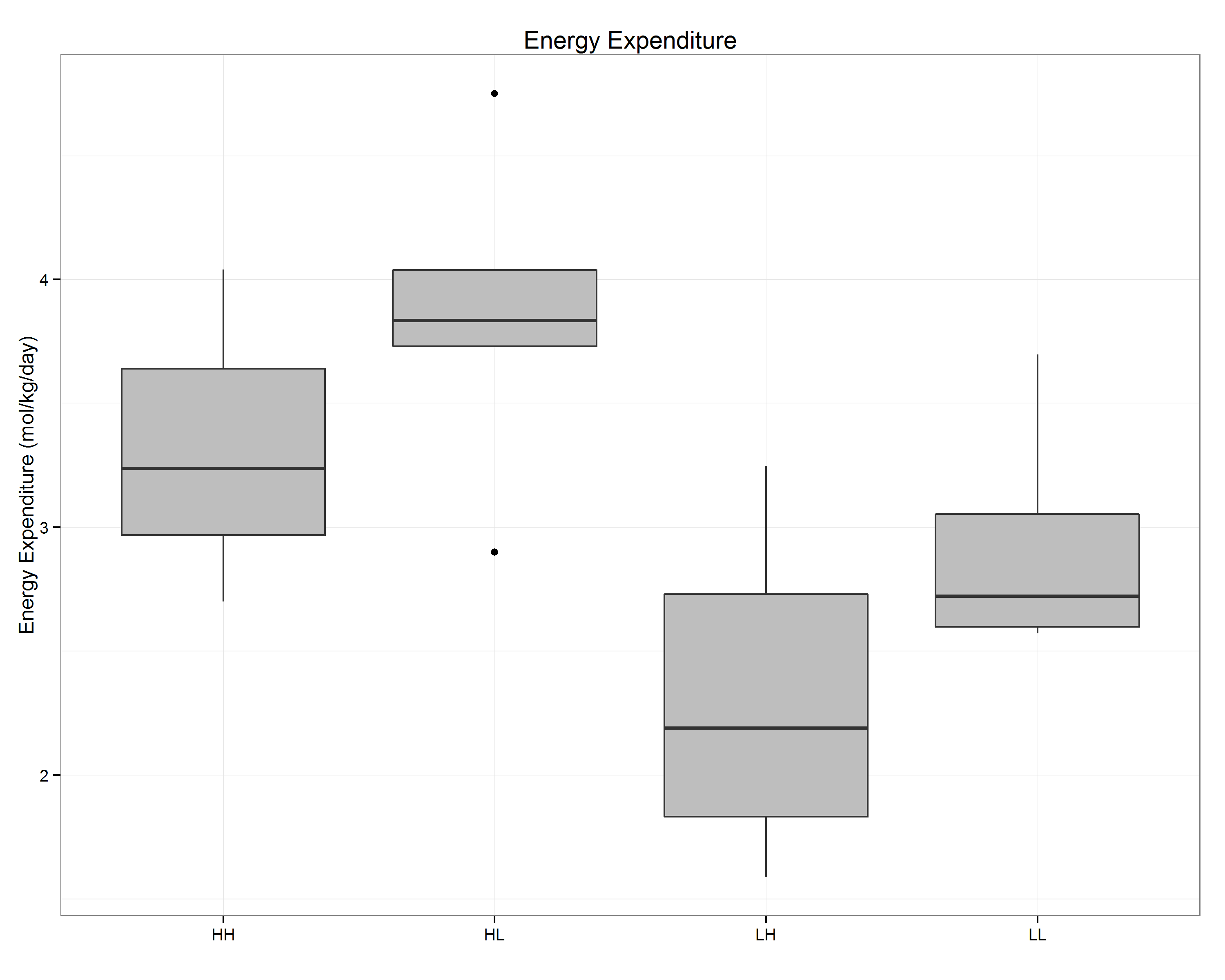

Supplement: Figure S5 — Energy expenditure in adult offspring is influenced by maternal diet. Energy expenditure (mol CO2 produced per day per kg of animal body weight) is increased in male offspring of mothers fed a high fat diet during pregnancy. (TIF) [file pone.0090335.s005.tif]

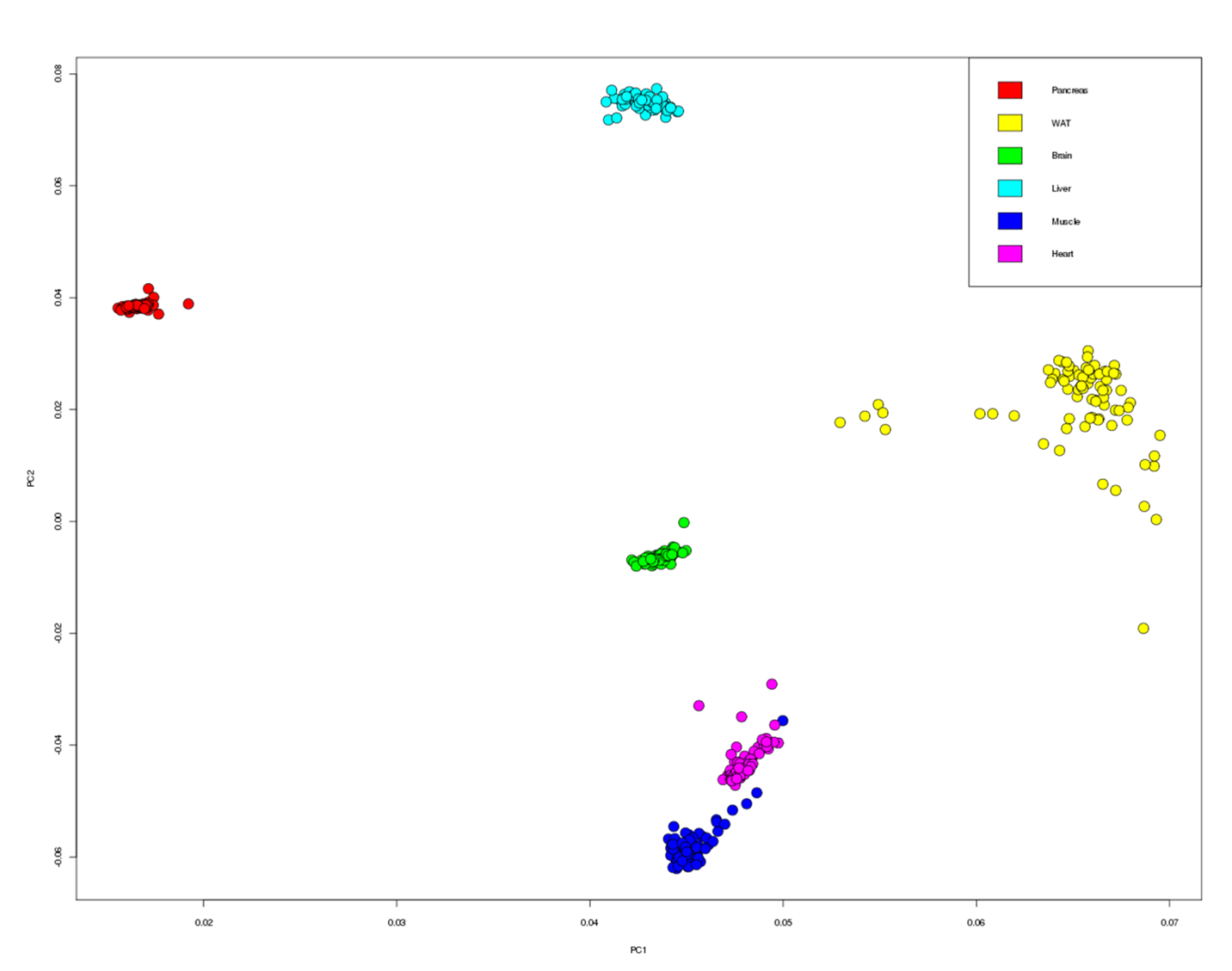

Supplement: Figure S6 — Principal component analysis of the gene expression patterns across tissues. Each dot represents one tissue from one male mouse. Red dots represent pancreas samples; light blue, liver samples; yellow, adipose tissues; green, brains; dark blue, muscle and pink, heart. (TIF) [file pone.0090335.s006.tif]

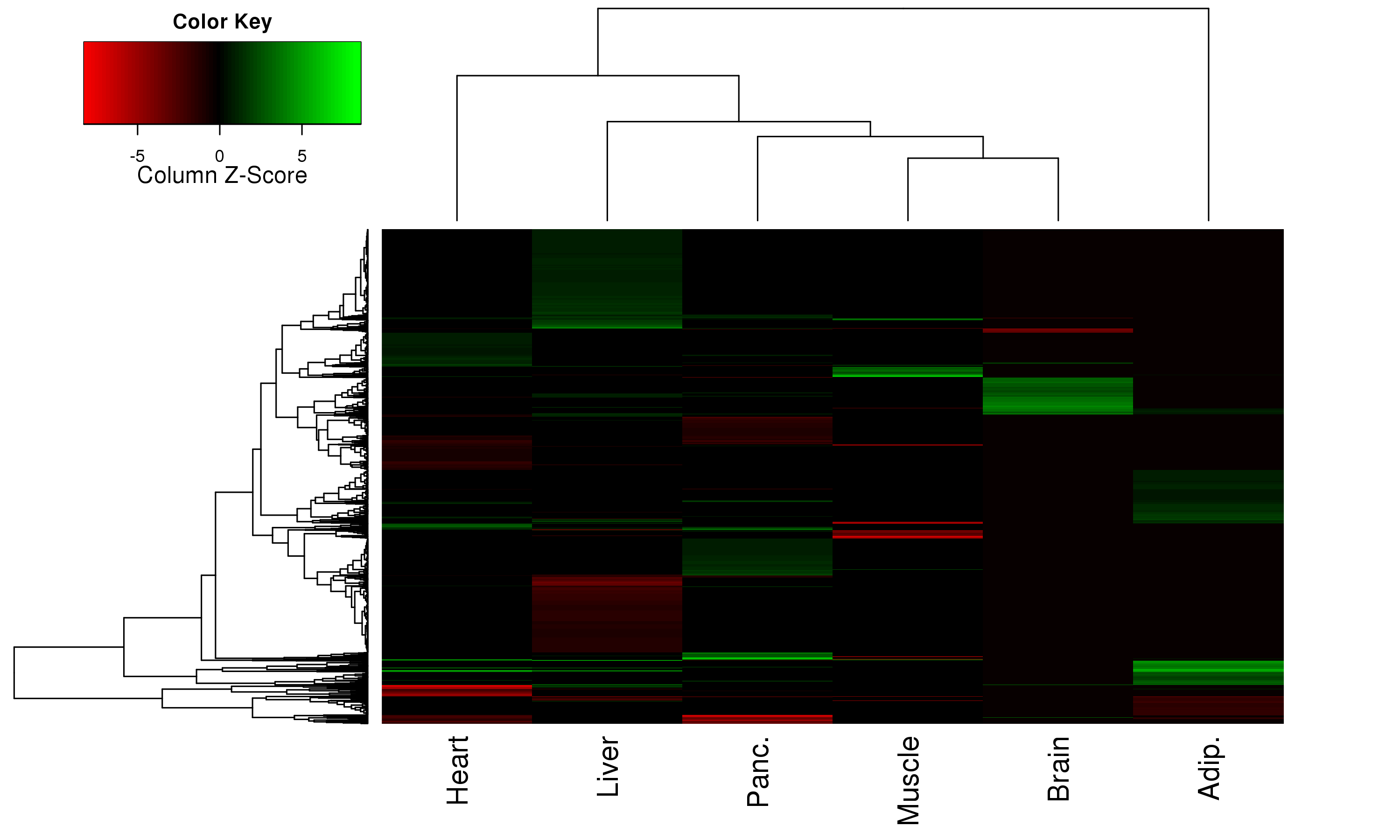

Supplement: Figure S7 — Heatmap of gene expression data for six tissues for the comparison of animals fed a high fat diet post-weaning, but from mothers fed a different diet. (TIF) [file pone.0090335.s007.tif]

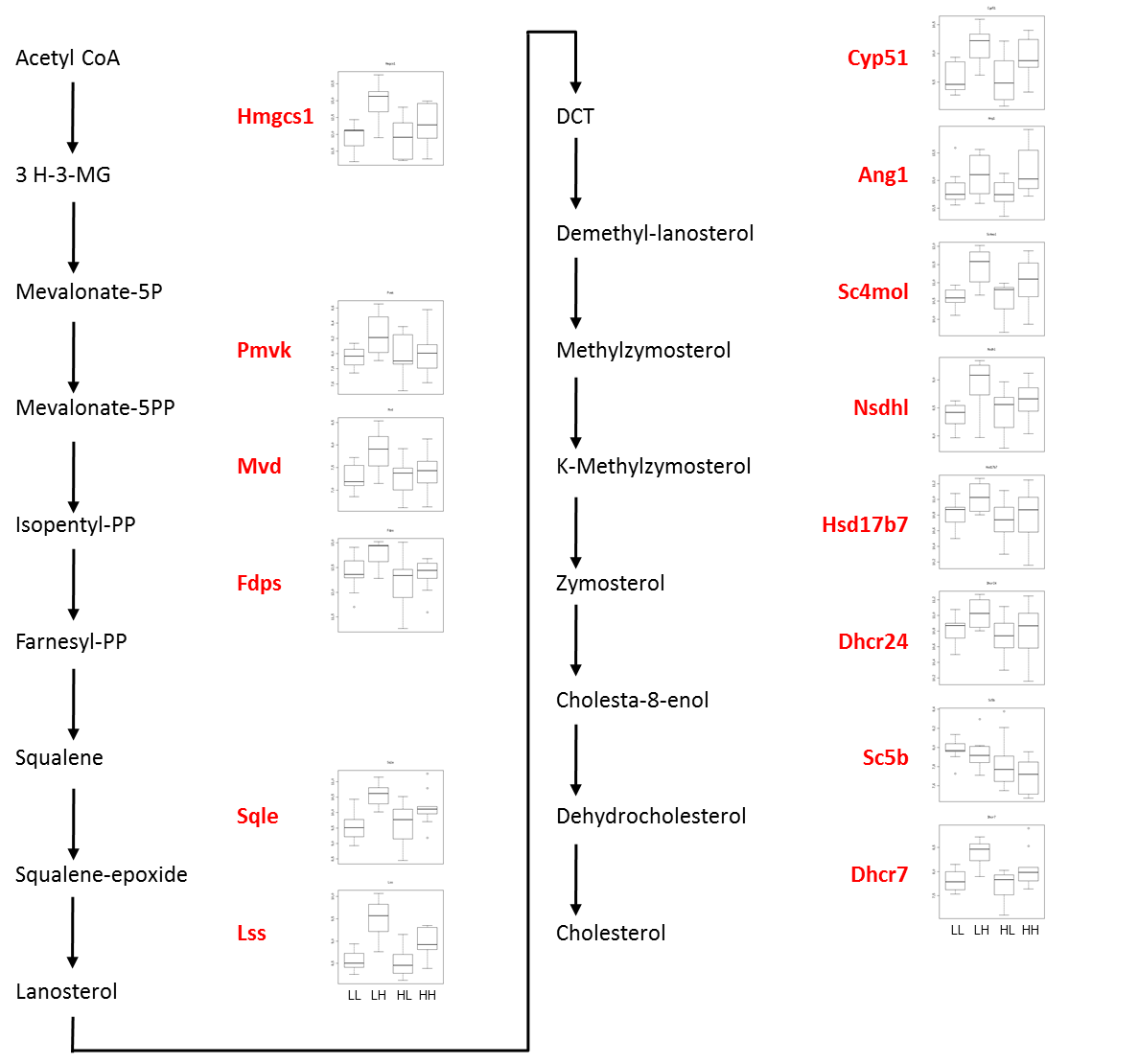

Supplement: Figure S8 — Liver gene expression level for 14 genes involved in cholesterol synthesis. See legend of Figure 3 for details. (TIF) [file pone.0090335.s008.tif]

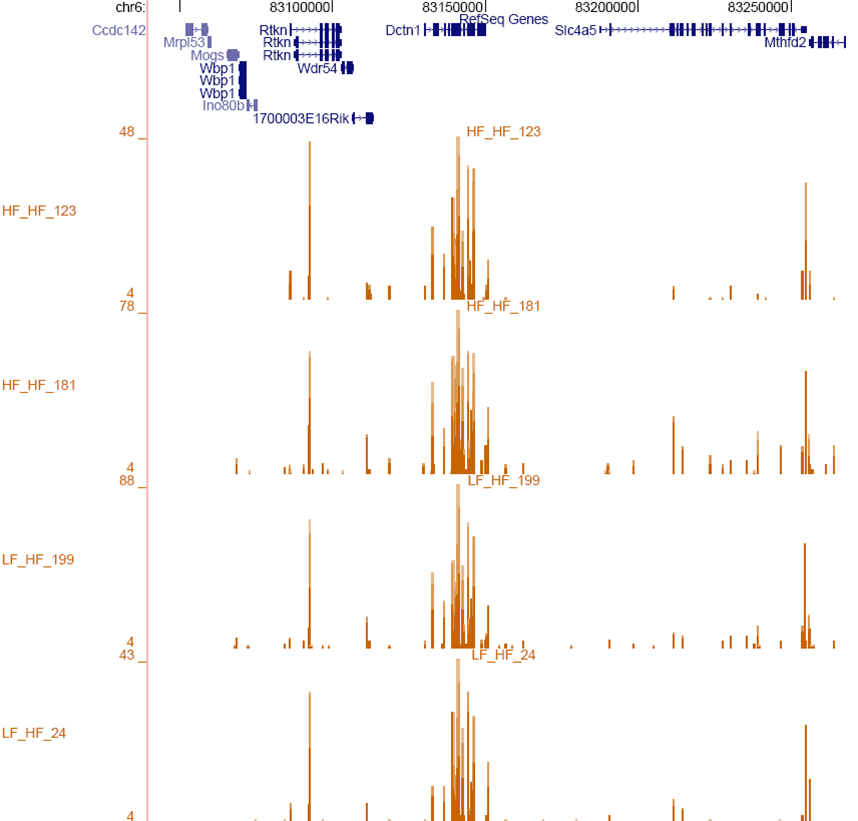

Supplement: Figure S10 — Comparison of the DNA methylation patterns obtained by MBD-GS across ∼200 kb in four liver samples. The figure shows the DNA methylation patterns across 200 kb of chromosome 6. The top track shows in blue the genomic coordinates of genes based on the UCSC annotation. The next tracks show the methylation patterns observed from MBD isolation and genome sequencing for two liver samples from males born from HF fed dams (top two tracks) and two liver samples from males born from LF fed dams (bottom two tracks). Each brown vertical line display the number of reads obtained per 100 bp window for each sample. (TIF) [file pone.0090335.s010.tif]

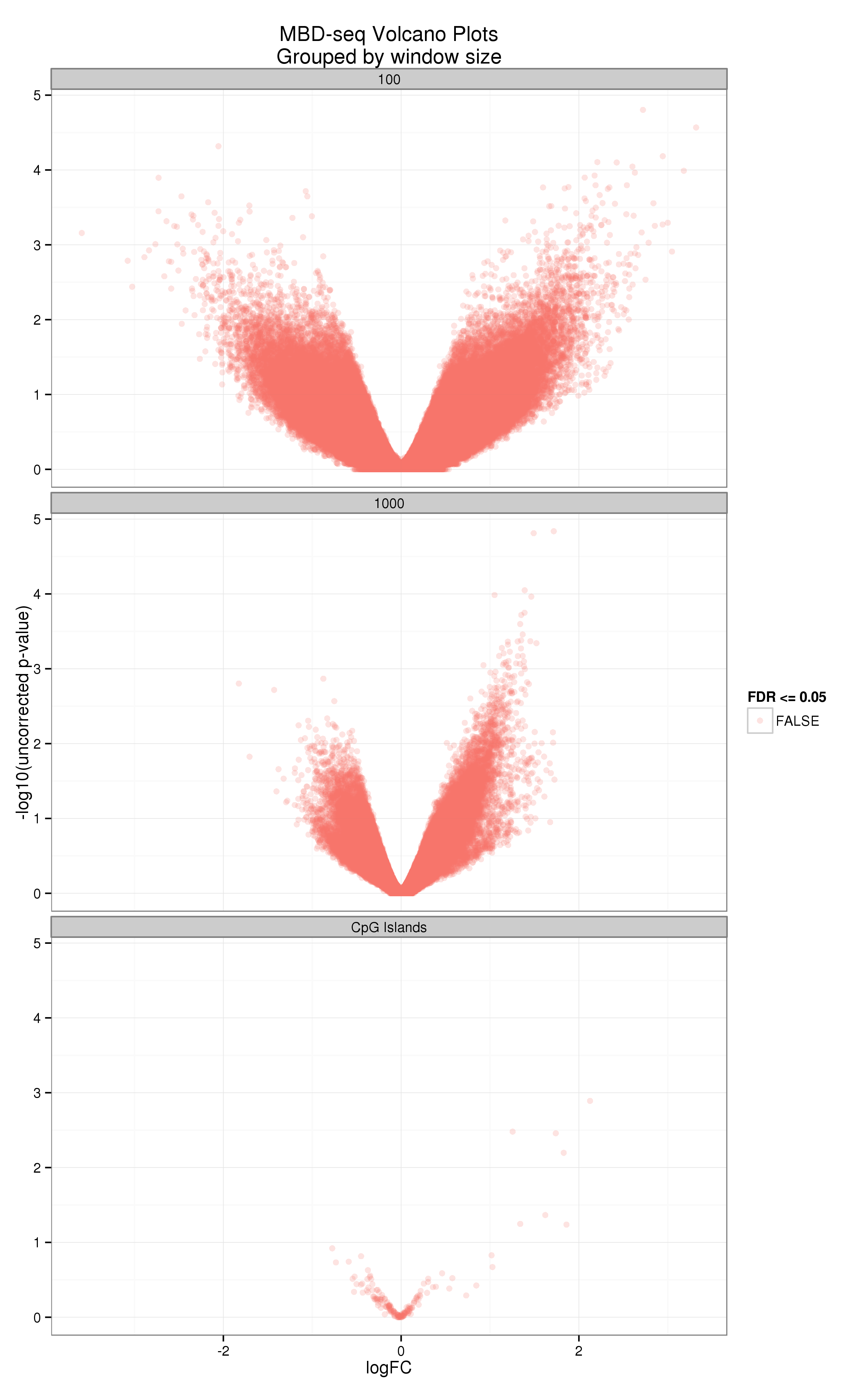

Supplement: Figure S11 — Volcano plots of MBD-GS results. The volcano plots show the fold change in MBD read counts (x-axis) and associated p-value (–log10(uncorrected p-value, y-axis) for each tested genomic locus (window sizes are indicated above the volcano plots). After FDR correction, no windows were significantly differentially methylated. (PNG) [file pone.0090335.s011.png]

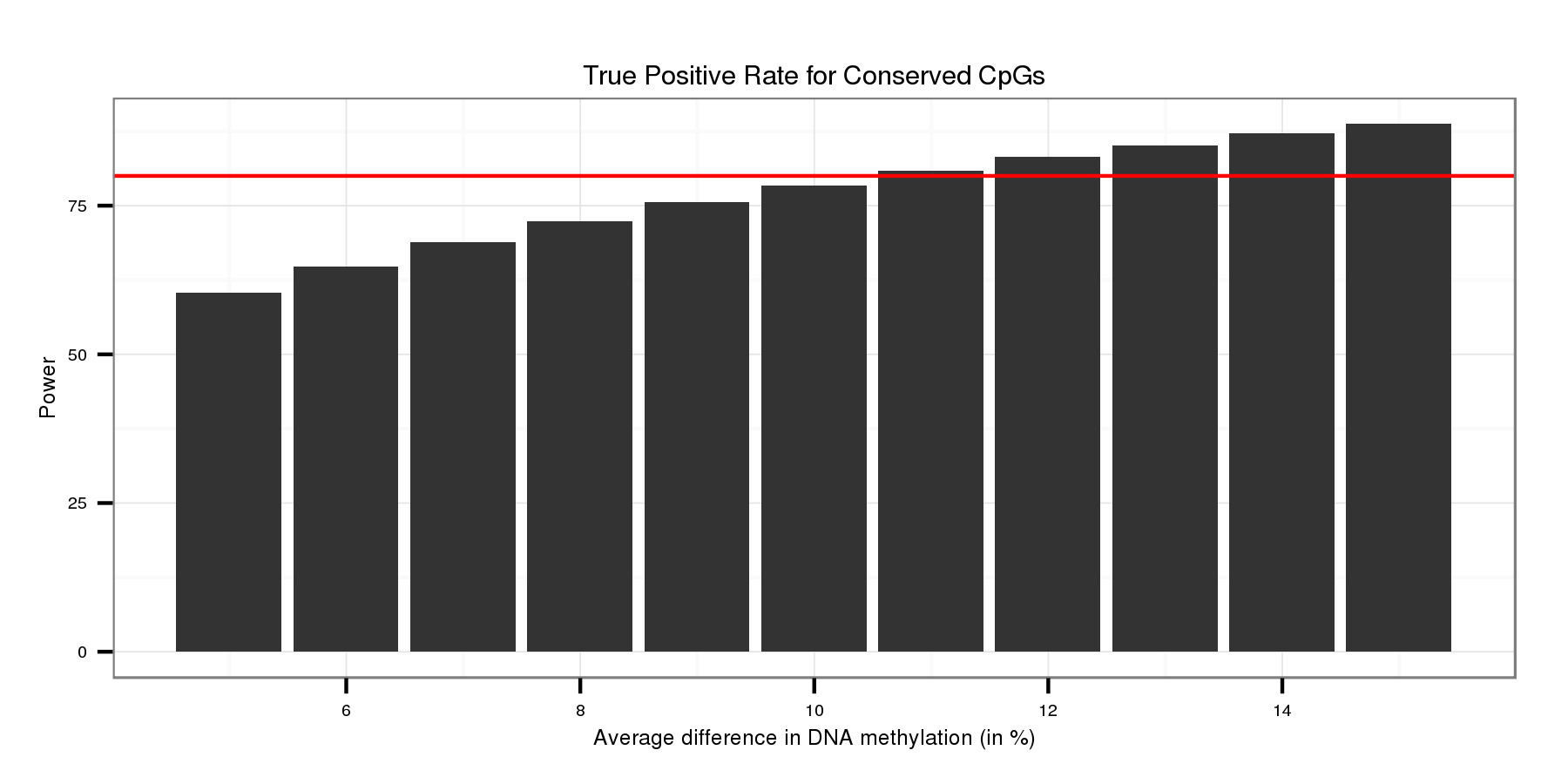

Supplement: Figure S12 — RRBS Power Analysis. The figure shows the statistical power (y-axis) to detect a given difference in DNA methylation (x-axis) at conserved CpGs. To estimate power, we randomly sampled means and standard deviations from the RRBS data generated for the HH group (n = 10) and simulated RRBS data for two groups (each n = 10) with a fixed average difference in DNA methylation (x-axis). 10,000 simulations (i.e. 10,000 random selections of mean and SD) were performed for each fixed difference in methylation and the percent of significant tests (p<0.05) calculated. Only CpGs with low DNA methylation variability within group (lowest 10% of standard deviation) were used in this analysis (the results are qualitatively similar using the entire dataset). The red line represents 80% power. (TIFF) [file pone.0090335.s012.tiff]

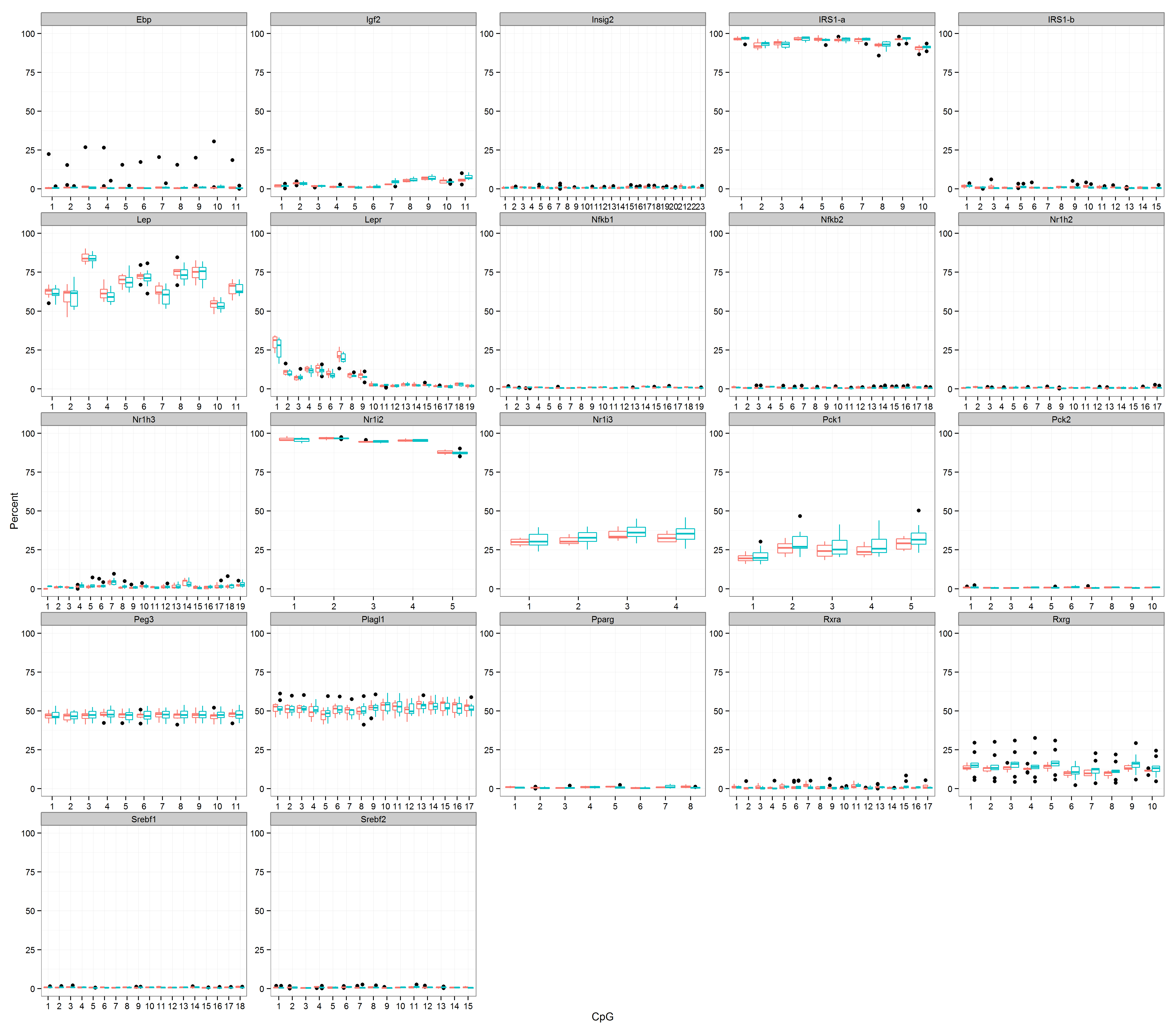

Supplement: Figure S13 — DNA methylation patterns at 22 loci using bisulfite sequencing. The figure shows the average cytosine methylation of each CpG of 22 selected loci after bisulfite sequencing of 20 liver sample from 9-week-old offspring fed HF after weaning and born from LF fed dams (in blue, n = 10) or HF fed dams (in red, n = 10).Each plot corresponds to a different locus. The y-axis shows the average DNA methylation (for 0 to 100%). The x-axis shows each CpG sequenced in a given locus. No difference remains significant after correction for multiple testing. (TIF) [file pone.0090335.s013.tif]
